# Production and therapeutic use of astaxanthin in the nanotechnology era

**DOI:** 10.1007/s43440-023-00488-y

**Published:** 2023-05-13

**Authors:** Karim Abdelazim, Amr Ghit, Dina Assal, Neamat Dorra, Nehad Noby, Sherine N. Khattab, Shaymaa Essam El Feky, Ahmed Hussein

**Affiliations:** 1 Department of Biotechnology, Institute of Graduate Studies and Research, Alexandria University, Alexandria, Egypt; 2 Department of Medicine and Aging Sciences, “G. d’Annunzio” University of Chieti-Pescara, Chieti, Italy; 3 Center for Advanced Studies and Technology, “G. d’Annunzio” University of Chieti-Pescara, Chieti, Italy; 4 Department of Biology, Biotechnology Program, American University in Cairo, Cairo, Egypt; 5 Department of Microbiology and Immunology, Faculty of Pharmacy, Sinai University—Kantara Branch, Ismailia, Egypt; 6 Chemistry Department, Faculty of Science, Alexandria University, Alexandria, Egypt; 7 Radiation Sciences Department, Medical Research Institute, University of Alexandria, Alexandria, Egypt

**Keywords:** Astaxanthin, Cancer, Chemotherapy, Antioxidants, Nanoparticles, Drug delivery, Green Chemistry

## Abstract

**Abstract:**

Astaxanthin (AXT) is a red fat-soluble pigment found naturally in aquatic animals, plants, and various microorganisms and can be manufactured artificially using chemical catalysis. AXT is a xanthophyll carotenoid with a high potential for scavenging free radicals. Several studies have investigated AXT efficacy against diseases such as neurodegenerative, ocular, skin, and cardiovascular hypertension, diabetes, gastrointestinal and liver diseases, and immuno-protective functions. However, its poor solubility, low stability to light and oxygen, and limited bioavailability are major obstacles hindering its wide applications as a therapeutic agent or nutritional supplement. Incorporating AXT with nanocarriers holds great promise in enhancing its physiochemical properties. Nanocarriers are delivery systems with several benefits, including surface modification, bioactivity, and targeted medication delivery and release. Many approaches have been applied to enhance AXT’s medicinal effect, including solid lipid nanoparticles, nanostructured lipid carriers (NLCs) and polymeric nanospheres. AXT nano-formulations have demonstrated a high antioxidant and anti-inflammatory effect, significantly affecting cancer in different organs. This review summarizes the most recent data on AXT production, characterization, biological activity, and therapeutic usage, focusing on its uses in the nanotechnology era.

**Graphical abstract:**

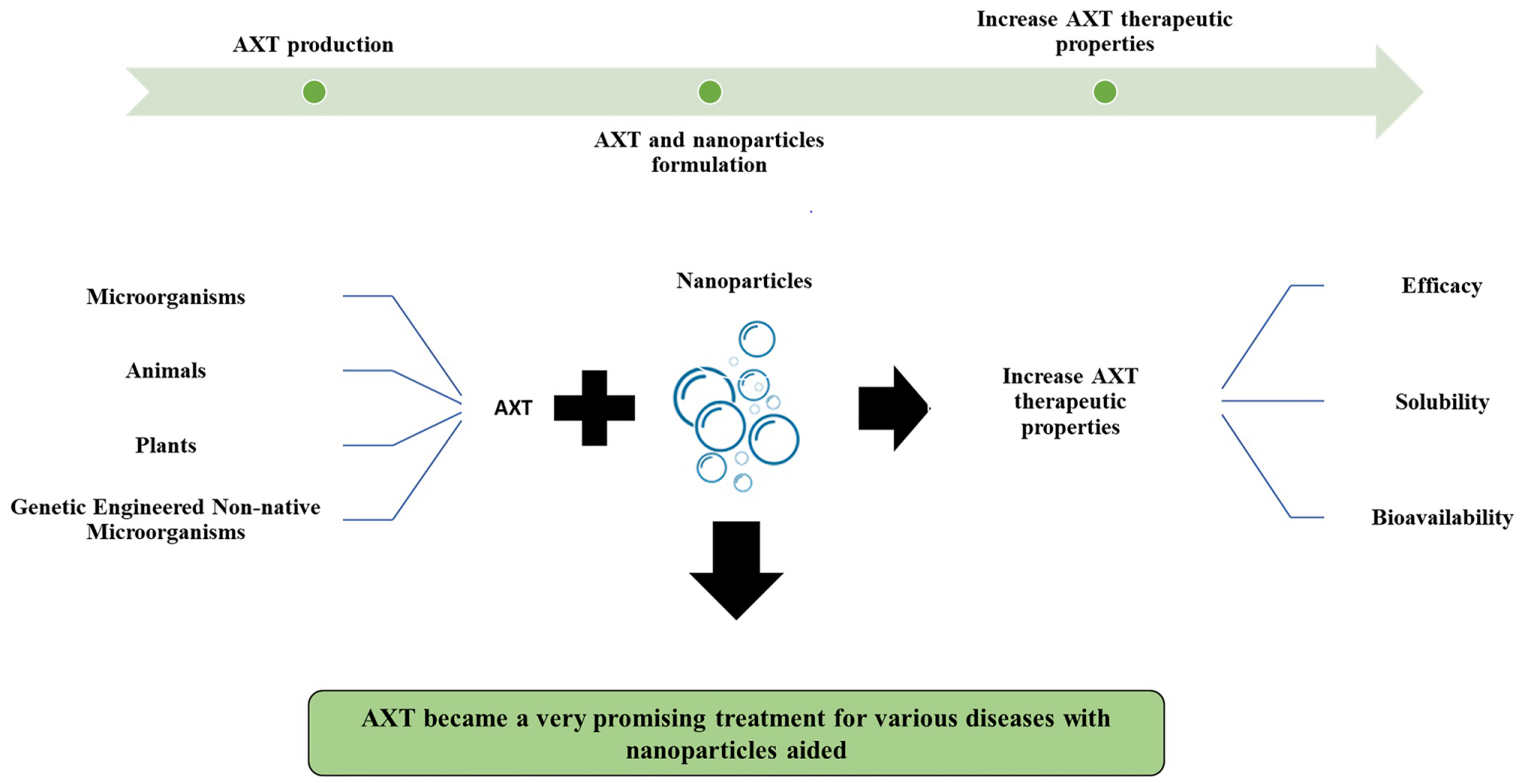

## Introduction

Astaxanthin (AXT) (3,3′-dihydroxy-β-carotene-4,4′-dione) is a xanthophyll carotenoid with a molecular mass of 596.85 Da. It could be chemically synthesized or found naturally in plants and some species of microorganisms like bacteria, yeast, and algae. It is used as feed in aquacultures to impart the characteristic red–orange color of aquatic animals such as crabs, shrimp, salmon, and trout [1]. The US Food and Drug Administration (FDA) has approved using AXT as a food color [2]. Due to the unknown effects of synthetic AXT on human health, in the long run, natural AXT is preferred in the food, pharmaceutical, and cosmetic industries. The green alga *Haematococcus pluvialis* is considered the primary source of AXT for human and animal consumption [3, 4]. The use of AXT as a nutritional supplement or medicine is growing rapidly. The global market of AXT is expected to reach 398.8 million $ by 2027, as stated by Grand review research [5]. AXT has several beneficial therapeutic properties due to its high antioxidant properties and is considered a strong candidate for treating numerous disorders. AXT intake can treat or lower the risk of several illnesses, including anti-aging and anticancer, besides providing neuroprotective benefits. However, AXT’s poor bioavailability, low stability, and solubility severely limit its use as a medicinal agent in clinical practice [6]. This review provides our current understanding of AXT sources, characterization, biological activity, therapeutic use, and how bioavailability problems can be solved using different nanoparticle technologies for future therapeutic applications.

## Sources of AXT

There are multiple different sources from which AXT can be produced, including microbial, plant, and animal sources (Table 1). The green microalga *Haematococcus pluvialis* is one of the best microbial sources of natural AXT, accumulating to 38% at the dry weight foundation [7, 8]. The first strain applied in the industrial production of AXT is *Xanthophyllomyces dendrorhous. I*t is one of the significant AXT-producing yeast; it contains roughly 0.2–0.5 mg/g dry cell weight (DCW) carotenoids, (40–95) % of which are AXT [9].

**Table 1:** Sources of AXT

Source	Example	References
Microorganisms	The natural microbial sources of AXT are algae *(Haematococcus pluvialis and Chlorella zofingiensis, Chlorococcum, Neochloris wimmeri, Enteromorpha intestinalis, and Ulva lactuca, Catenella repens*), bacteria *(Paracoccus spp. and Brevundimonas spp*.), fungi (the *basidiomycetous* yeast, *Xanthophyllomyces dendrorhous*, also known as *Phaffia rhodozyma*) and protist (*Aurantiochytrium sp., Thraustochytrium sp*)	[5, 9, 11]
Animal	Birds like flamingos and quails, as well as marine creatures like salmon, shrimp, lobster, crab, and krill, contain AXT in nature	[10, 11, 12]
Plant	*Adonis aestivalis* and *Artemisia annua*	[13, 14]
Genetic engineered non-native organisms	Examples of genetically modified organisms that make AXT include *Escherichia coli, Saccharomyces cerevisiae, Corynebacterium glutamicum, Kluyveromyces marxianus, Synechococcus sp., and Yarrowia lipolytica*	[9]

AXT: Astaxanthin: PH: Potential of hydrogen; PLA: Poly-lactic acid; PLGA: Poly-lactic-co-glycolic acid

AXT is found in many animals, including birds like flamingos and quails and marine creatures like salmon, shrimp, lobster, crab, and krill [10]. AXT accounts for 74% to 98% of all pigments in the crab shell. Krill oil contains significant AXT (0.1–15 mg/ml) depending on the processing technique [11]. The animals, conversely, do not produce AXT; they obtain it from natural AXT producers found in their diets. However, the animals do not generate AXT; they obtain it from the natural AXT producers found in their diets [12].

Many flowering plants in the genus Adonis also contain AXT, and their bright colors help attract pollinators. Among plants, *Adonis aestivalis* is the largest source of AXT; about 1% of their dry matter comprises AXT [13, 14].

Synthetic AXT differs in its makeup from natural AXT. In synthetic AXT, there are various stereoisomers, some of which do not occur naturally, have poor bioavailability, and are less technologically stable. EC Regulation No. 1925/2006 on vitamins, minerals, and other food additives prohibits synthetic AXT in food. It also is not generally considered safe (GRAS) status in the US. However, it appears that synthesized AXT might be dangerous for people or animals. It is frequently used in the feed industry, particularly aquaculture, as a pink and red color [13].

Applying genetic engineering and synthetic biology strategies makes obtaining free AXT with high purity in non-native microorganisms relatively easy and increases the production of AXT from native organisms [12]. For instance, random mutation and overexpression of AXT biosynthetic genes of *Paracoccus sp* have helped raise the productivity o 480 mg/L in fed-batch fermentation [15]. Also, the optimized strain of *Yarrowia lipolytica* generated 0.3 g/L of AXT in fed-batch cultivation with cellular content of 6 mg/g dry cell weight (DCW) [16].

## AXT production challenges

AXT production by *H. pluvialis*, microalgae, faces several economic and laboratory challenges [17].

The bioprocess for producing AXT is complex and challenging to scale up due to the need for two-stage cultivation with a high-intensity light. Additionally, microalgae have a slow growth rate, making the production phase relatively long and more susceptible to contamination. Non-natural producers also face challenges due to the inefficiency of heterologous enzymes and pathway complexity, which can lead to the accumulation of structurally similar intermediate carotenoids [12].

Genetic engineering of carotenoid metabolic pathways has been used to increase yields in several species, but this approach presents specific challenges, including the need for detailed knowledge of carotenoid biosynthesis pathways, availability of nuclear and chloroplast genomes, and knowledge of cellular enzyme localization [18].

Another significant challenge in microalgae production is the high cost of other food commodities. The cost of producing microalgal biomass depends on the reactor’s location and the biomass’s overall quality. In Europe, the cost of producing microalgal biomass is around 10–50 €·kg^−1^, which is much higher than that of common food ingredients like soya, wheat, and rice [19].

Thus, addressing these challenges is crucial for the cost-effective and efficient production of AXT and other microalgae-based products.

## Characterization of AXT

### Primary structure and isomers of AXT

**Fig. 1:**
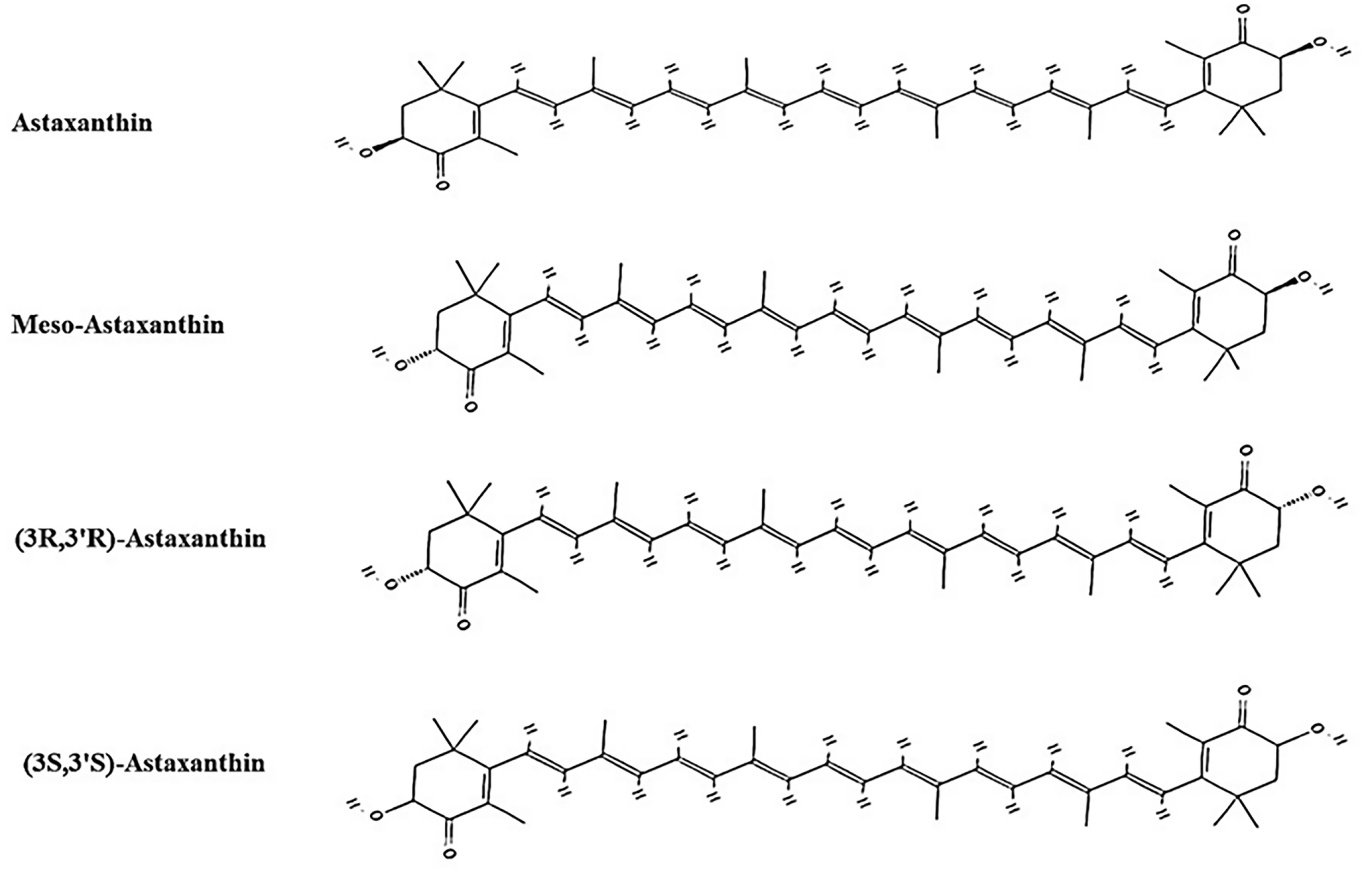
Chemical structure of astaxanthin and its three configurational isomers. AXT (PubChem CID: 5281224). Meso-AXT (3S,3'R) AXT or 3R,3'S-AXT (PubChem CID 12358422). 3R,3'R-AXT or (3R,3'R)-AXT (PubChem CID: 12358421). (3S,3'S)-AXT (PubChem CID: 5281224). AXT: Astaxanthin

**Fig. 2:**
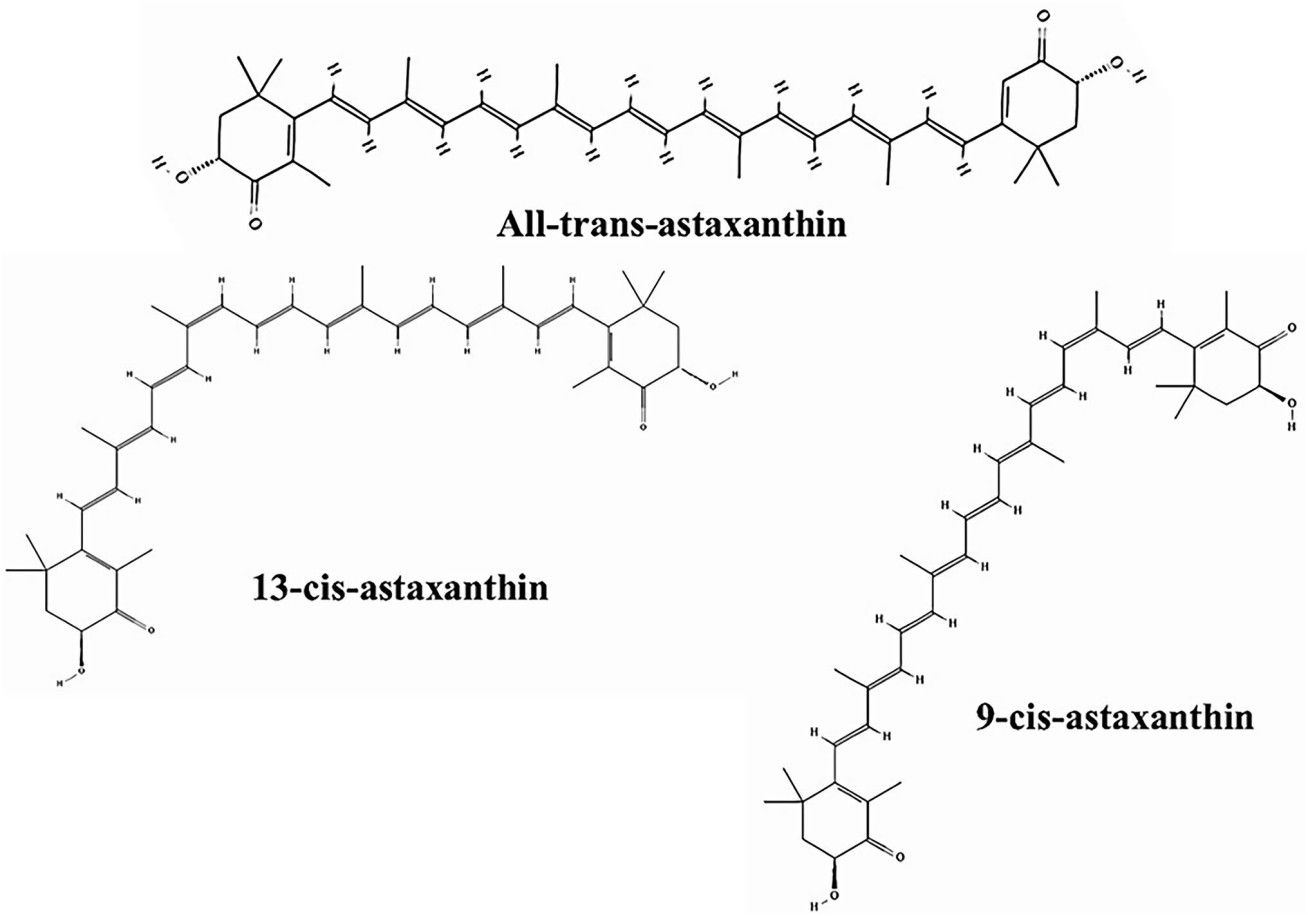
AXT trans and cis structure. All-trans (PubChem CID: 118989688), 9-cis (PubChem CID: 91827091), and 13-cis-AXT (PubChem CID: 91827159). AXT: Astaxanthin

Metabolically active AXT (3,3′-dihydroxy-β, β-carotene-4,40-dione) is a xanthophyll carotenoid; with a molecular formula C40H52O4 and a molar mass of 596.84 g/mol [1]. Like most known carotenoids, AXT comprises a C40 skeleton of linked isoprene units. The system consists of 11 conjugated double bonds, determining the characteristics red color of AXT and is responsible for its antioxidative activity. The structure comprises two β-ionone–terminal rings joined by a linear polyene chain. Both terminal rings contain a hydroxyl group (OH) localized at 3 and 3′ positions of the two asymmetric carbons and two keto groups (=O) located at carbons C4 (Fig. 1). The structure’s polar and non-polar groups enabled AXT to express its antioxidative activity in the lipid system. The hydrophobic polyene chain can fit inside the membrane’s lipid bilayer, while its polar groups are oriented near the membrane surface, constituting a unique structure among known carotenoids. Based on the configuration of the hydroxyl group of the asymmetric carbon C3, three different stereoisomers are formed: (3S,3′S), (3R,3′R), and (3R,3′S) (Fig. 1). The abovementioned isomers differ in bioavailability, physicochemical and biological properties, and their occurrence ratio in nature depends on the source where they are obtained. In addition, the hydroxyl group can react with fatty acids, such as palmitic, oleic, stearic, or linoleic acid, to form mono- or diesters, accordingly, which increases its solubility in the cell and makes it more stable to oxidation [5, 20]. AXT also exists as trans and cis (E and Z) geometrical isomers, depending on the configuration of the double bonds in the polyene chain. All-trans AXT is the dominant isomer, although at least two cis-isomers (9-cis and 13-cis) also occur in nature, depending on the host species and body part. In addition, they are found in synthetic preparations [21] (Fig. 2).

Although AXT possesses a high pharmacological potential, it has few applications in the clinical setting due to poor drug stability [22]. The molecular structure of carotenoids contains C=C double bonds, which render the molecule vulnerable to air oxidation and photo-oxidation. These elements collectively can transform them into a different stereoisomer (with trans–cis isomerization) [1]. Furthermore, they increase the molecule’s sensitivity to thermal degradation and worsen its water solubility [5] Therefore, creative approaches are needed to overcome the limits of this intriguing natural active compound, thus allowing its exploitation in the therapeutic field.

## The safety profile of AXT

According to various studies and experiments, AXT has been demonstrated to have a favorable safety profile. Its global market value has risen significantly over the years. Safety testing of an AXT-enriched extract showed no clinically significant changes in blood pressure, hematology, or blood chemistry parameters in healthy adults at 6 or 20 mg daily for eight weeks and four weeks, respectively. Furthermore, a dose of 8 mg daily for three months did not cause gastrointestinal tract distress or other side effects [23].

Studies on oral administration of natural AXT have not reported any adverse effects, even at high doses of up to 1240 mg/kg/day in rats for 90 days. Clinical safety studies have shown that AXT is safe compared to the placebo, even at low doses of up to 12 mg and high doses of up to 100 mg per day [24].

Numerous experiments, including acute toxicity, mutagenesis, transgenicity, fetal toxicity, and reproductive toxicity, have confirmed the safety of AXT [25].

Furthermore, there has been an exponential increase in the number of studies examining the health, beauty, and safety of AXT, and a review of 87 AXT clinical trials involving humans found no serious adverse effects [26].

Overall, the safety profile of AXT appears to be excellent based on the available evidence.

## Pharmacokinetics and bioavailability of AXT

**Fig. 3:**
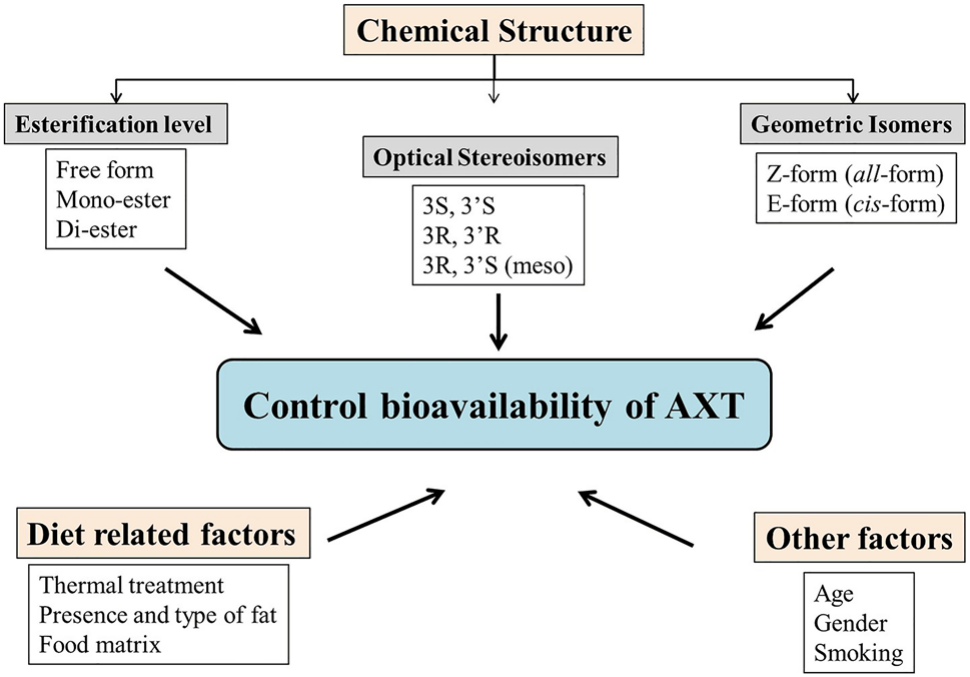
Factors affecting AXT bioavailability. AXT: Astaxanthin

Of note, the bioavailability of AXT is affected mainly by its chemical structure [27, 28] (Fig. 3). The absorption of AXT in organisms takes place through its passive diffusion alongside fatty acids into the intestinal epithelium. After ingestion, AXT is combined with bile acid in the intestine, forming micelles. Intestinal mucosal cells absorb AXT from micelles, which are then incorporated into chylomicrons and carried to the liver. Lipoproteins then transport AXT to various tissues. AXT protects the mitochondrial redox state and functions by inserting itself into lipid bilayers and maintaining membrane integrity. It is believed that carotenoids are finally transported to the tissues by circulating, where they are incorporated into very low-density lipoproteins (VLDL), low-density lipoproteins (LDL), or high-density lipoproteins (HDL) [28, 29].

In their study, Odeberg et al. administered 40 mg of AXT to healthy male volunteers to determine AXT’s pharmacokinetic parameters. Depending on the source, this preparation was either a commercial dietary supplement or a lipid-based formula. The bioavailability of lipid-based formulations was significantly higher than reference formulations (*p* < 0.05) [30]. More effective methods, such as nanoformulations and targeted therapies, are needed to improve AXT’s bioavailability. The pharmacokinetic parameters of AXT-containing formulations were studied in animal models, but few human studies have been reported. In a study by Singh et al., they administered macro, nano, and oil solutions to rats. They investigated the correlation between the formulation’s size and AXT’s tissue distribution. Their study reported that nano-sized emulsions improved the distribution of AXT in tissues and enhanced its bioavailability [31].

According to a study of spleen contents, spleens had the highest AXT concentrations (1673.28 ± 99.86 μg/g wet weight). These results may illuminate why AXT has a prominent effect on the immune system. The kidney is the primary excretory organ for AXT, following the spleen. In descending order, the spleen, kidney, heart, lung, and liver had the highest total concentrations [32, 33].

It is worth noting that there is a vast difference in carotenoids’ bioavailability, which varies from 10 to 50% in humans. AXT can be absorbed through intestinal epithelial cells and released into lymph as chylomicrons due to its low solubility and dispersibility in gastrointestinal fluids [29]. Evidence suggests that various parameters influence carotenoids’ absorption. These parameters can be categorized into chemical properties and diet- and non-diet-related parameters (such as age, gender, fat type, and fat content). Carotenoid bioavailability is determined by three types of chemical complexity: chemical structure, optical stereochemistry, and geochemistry of the isomeric form. In addition, food consumption (especially fatty food) increases AXT absorption, while smoking significantly reduces the half-life of AXT’s elimination [27, 33].

AXT’s poor solubility and stability limit its use in functional food, health products, and medicines [34]. One approach for improving AXT’s in vitro release is to apply novel formulations with functional ingredients. Although these formulations included molecular complexes, microemulsions, liposomes, solid nanoparticles, biopolymer particles, and microgels, pharmacokinetic studies did not show enough AXT to reach a high blood concentration. Hence, Nanopreparations can be an appropriate drug delivery form, increasing insoluble drugs’ bioaccessibility [35].

### Improving AXT bioavailability

AXT is a carotenoid with potent antioxidant and anti-inflammatory activities that result from its highly unsaturated molecular structures. It could be a good candidate for treating many diseases, possesses many valuable therapeutic functions such as anti-aging, anticancer, and has a neuroprotective effect [7]. However, AXT’s limited stability and solubility lead to poor absorption, significantly limiting its therapeutic use in clinical practice.

**Fig. 4:**
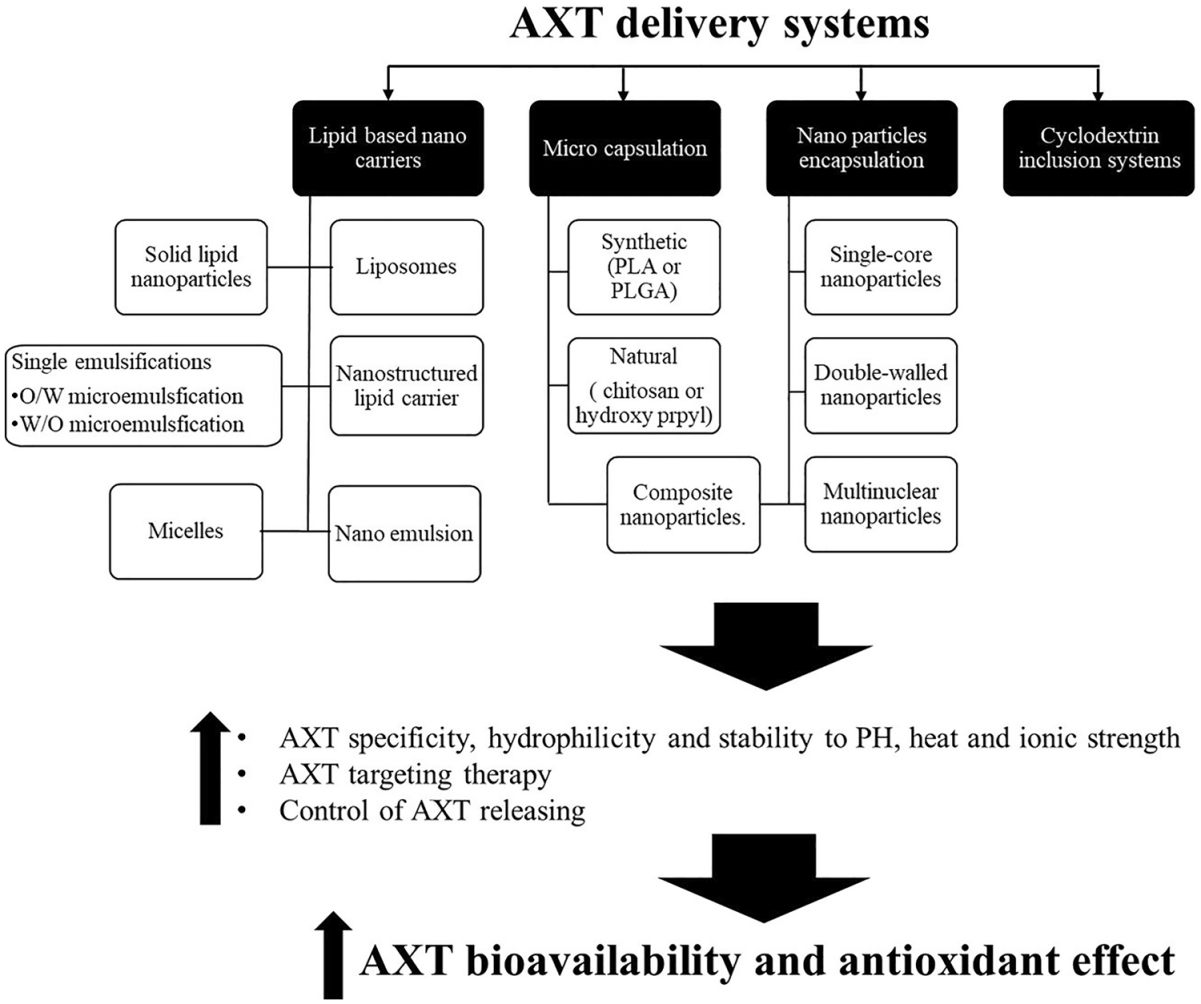
Role of AXT delivery systems in improving the bioavailability and antioxidant effect. AXT: Astaxanthin

Notably, AXT delivery systems, such as lipid-based nanocarriers, macro capsulation, nanoparticles, and cyclodextrin inclusion systems, improve AXT bioavailability and antioxidant effect [36, 37] (Fig. 4).

Recently, nanomedicine, particularly solid lipid nanoparticles (SLNs), has been attracting considerable attention due to its potential advantages for enhancing pharmacokinetics and the pharmacodynamic activity of naturally derived constituents. SLNs are colloidal carriers made from solid biodegradable lipids stabilized by surfactants, with a mean diameter ranging from 50 to 1000 nm [38] presenting several advantages over other colloidal carriers (liposomes and polymeric nanoparticles) such as higher drugs, absence of toxicity, easy large-scale production, and the possibility of lyophilization [39].

These nanoparticles’ composition and characteristics make them ideal for carrying/delivering sensitive bioactive compounds, protecting them against chemical degradation and facilitating their application in various administration routes [40].

The encapsulation of drugs into these nanocarriers increases their solubility, stability, cell uptake, specificity, tolerability, and therapeutic index [41, 42].

Unfortunately, the use of SLNs through systemic administration is limited by the opsonization process; an important mechanism used by the immune system to fight pathogens, which is responsible for their short half-life (3–5 min) after intravenous administration [43, 44].

One of the most used strategies to prolong these carriers’ systemic residence is modifying the nanoparticle surface to achieve ‘stealth’ systems to overcome the defense line represented by macrophages through a biomimetic mechanism. A strategy used to realize ‘stealth’ systems; is to coat the nanoparticle surface with surfactants, such as poloxamers. This hydrophilic coating modifies the SLN biodistribution, improving blood circulation times and deposition in non-RES (reticuloendothelial system) organs [45, 46].

Among the different surfactants used to modify particle distribution, polysorbate 80 (p80) is one of the most effective surfactants for enhancing concentration in the central nervous system (CNS).

The remarkable scavenging activity of AXT is due to its chemical structure which traps radicals in the polyene chain (Fig. 1). It is a highly lipophilic molecule. However, the presence of two terminal hydrophilic groups determines a slight solubility in water.

It’s dual nature and small size allow it to permeate cell membranes readily. It is a carotenoid that carries out its antioxidant and anti-inflammatory actions at the level of the eyes, brain, and CNS, as it can cross the blood–brain and blood-retinal barriers [47].

## Therapeutic uses of AXT

### AXT’s role as an antioxidant and anti-inflammatory

The chemical structure of AXT makes it a potent antioxidant with the capacity to scavenge free radicals, protect the mitochondria, reduce inflammation, and prevent glycation [48]. It can quench single oxygen and significantly protect against oxidative stress and age-related disorders. Also, AXT cooperates to protect against lipid peroxidation [49].

The AXT reduces the O^2−^ level in the Lipopolysaccharide (LPS)-stimulated pro-monocytic, human myeloid leukemia cell line (U937). Treatment with AXT results in the antioxidant network’s functional recovery by reducing O^2−^ levels. Therefore, nuclear factor erythroid 2–related factor 2 (Nrf2) does not translocate into the nucleus, and there is not any evidence of increased expression and catalytic activity of heme oxygenase 1 (HO-1) [50].

Consuming AXT in the diet improves immune function, reduces inflammation, and is a biomarker for deoxyribonucleic acid (DNA) oxidative damage in young, healthy females. However, antioxidants generally show more significant physiologic modulation in cases with excessive amounts of oxidative stress and immuno-compromised individuals [32].

In dry eye disease (DED) models, AXT may suppress inflammation by downregulating the production of high-mobility group box 1 (HMGB1) and inflammatory cytokines, tumor necrosis factor-alpha (TNF-α), interleukin-1 (IL-1). So, AXT may be a viable natural derivative for ocular surface protection in DED [51].

AXT reduced inflammation in endotoxin-induced uveitis (EIU) in a dose-dependent manner. For instance, 100 mg/kg of AXT had an anti-inflammatory impact on the ocular comparable to 10 mg/kg of prednisolone. AXT may have an anti-inflammatory impact on the ocular by directly inhibiting the activity of the nitric oxide synthase (NOS) enzyme, which could also reduce the generation of nitric oxide (NO), prostaglandin E2 (PGE2), and TNF-α [52].

According to preclinical and clinical research, AXT’s anti-inflammatory and antioxidant properties slow the development of cardiovascular diseases as AXT reduces the buildup of cholesterol in foam cells and the development of atherosclerotic plaques. These qualities of AXT make it a prospective choice for the preventive and/or adjuvant treatment of cardiovascular diseases [53].

### AXT as an anticancer agent

According to in vitro research, AXT inhibits cell proliferation, arrests the cell cycle, and induces apoptosis to prevent cancer through various pathways. Additionally, AXT improved the sensitivity of tumor cells to treatment and reduced its adverse effects [54]. AXT’s antioxidative property prevents stress from impairing natural kill (NK) cell antitumor activity, which may help explain why it inhibits cancer spreading [55].

Different stereoisomeric AXT significantly inhibited human colon cancer (HCT116 and HT29 cells). As a result of the stereoisomeric AXT’s modulation of oncogenic signalling proteins, the suppression of cancer cells was linked to substantial cell cycle arrest and apoptosis. The findings contradicted the initial theory that the anticancer activity of AXT was linked to the structures mentioned earlier and suggested that hydroxyl at C3 and C3′ of the terminal ring structure may not be the primary factor that affects the anticancer activity of AXT. The structure of long conjugated chains, unsaturated ketone (C=O), and α-hydroxy ketones probably contribute to the anticancer effects of AXT stereoisomers [56].

Treatment with AXT is an efficient method for preventing breast cancer cells from proliferating and migrating. It has been consistently established that AXT can reduce several different cancers. These discoveries could lead to various medical studies that could influence how cancer is treated today [57].

AXT was discovered to increase radiosensitivity and trigger apoptosis in esophageal squamous epithelial cell carcinoma cells (LS-180 cells) by decreasing B-cell lymphoma 2 (*Bcl-2*), *CyclinB1*, cell division cycle 2 (*Cdc2*) and favoring Bcl-2 associated X-protein (*Bax*) expression. By upregulating the expression of the genes *Bax* and *Caspase-3* and downregulating the expression of the gene *Bcl-2*, it has been discovered that AXT induces apoptosis in LS-180 cells and inhibits the growth and multiplication of cancer cells [58].

### Brain protection and neuroprotective role

AXT is gaining considerable attention as a pharmacological agent for treating various neurological diseases such as Alzheimer’s disease (AD), Parkinson’s disease (PD), neuropathic pain (NP), depression, autism, aging, and injuries to the brain and spinal cord [59].

Experimental data from animal studies have demonstrated that AXT has neuroprotective effects. In Vascular dementia, AXT improves memory and protects against stroke and hypertension. Also, AXT provides brain protection because it can cross the blood–brain barrier. Earlier findings also reported reduced blood pressure in individuals with a daily dose of 6–8 mg [49]. Fassett et al. reported that a dose of 50 mg/kg of AXT oil lowered systolic and diastolic blood pressure in rats with metabolic syndrome suffering from spontaneous hypertension, suggesting that AXT improves cognitive functions and protects against aging in rats [49, 60].

Alzheimer’s is regarded as one of the most common chronic and severe neurodegenerative disorders. It is reported that the antioxidant AXT can fight Alzheimer’s disease. Previous results reported that supplementing Alzheimer’s disease Wistar rats’ model with AXT powder isolated from shrimp shells has greatly ameliorated cognitive functions. Also, it is reported that the synthetic formula of AXT’ docosahexaenoic-acid-acylated AXT diesters (AXT-DHA) ameliorated cognitive disorders in mice with amyloid protein precursor/presenilin-1 (APP/PSEN1) by reducing chronic neuroinflammation and oxidative stress [61].

More studies demonstrated the effectiveness of AXT in managing AD. For instance, Taksima et al. demonstrated that Wistar rat’s models expressing AD symptoms showed a significant improvement in cognitive abilities after consuming AXT powder isolated from shells of Litopenaeus vannamei shrimps. By measuring object recognition and plaque levels in Wistar rats, AXT significantly improved their spatial and non-spatial aspects of working memory and decreased neurodegeneration [62]. According to their study, AXT alleviates cognitive function and diminishes neurodegenerative symptoms in AD Wistar rats model induced by amyloid-β peptides [62, 63].

In treating NP, antidepressants, and anticonvulsants are the primary options. However, there is increasing demand for novel therapeutic alternatives for NP treatment that concurrently target multiple mediators contributing to overall NP pathogenesis with acceptable efficacy and safety [36].

According to Qiao et al., AXT or lithium chloride combined with omethoate significantly reduced depressive-like behavior in an omethoate-induced depressed mouse [64].

In a similar study, Ke et al. suggested that AXT may be used to prevent diabetes and depression co-morbidities. As an intraperitoneally administered dose of AXT (15 or 25 mg/kg), increased expression levels of various signaling molecules such as phosphorylated Adenosine 3′5’ Cyclic Monophosphate)-Response Element Binding protein (pCREB), phosphorylated Protein kinase-like endoplasmic reticulum kinase (pERK), phosphorylated protein kinase B (pAKT) and brain-derived neurotrophic factor (BDN) in the prefrontal cortex (PFC) in Streptozotocin (STZ)-induced diabetic rats and thus, ameliorated depression [65].

### Eye health

The beneficial effects of AXT on eye health are examined by earlier studies and reviewed elsewhere [33].

According to animal and human studies, AXT may promote eye health with promising clinical outcomes in ocular diseases such as age-related macular degeneration, diabetic retinopathy, glaucoma, and cataracts [66].

Growing evidence supports AXT’s diverse effects on preventing and treating eye diseases. The diseases affect the anterior and posterior poles of the eye. Food micronutrients and nutraceuticals may act as bioactive compounds because they are physiological constituents of human tissues. They contribute to eye health through several metabolic pathways to maintain homeostatic balance in eye tissues. AXT is characterized by a broad spectrum of properties, such as antioxidant and anti-inflammatory activities, and its ability to regulate metabolism.

Furthermore, natural carotenoid treats chronic subclinical inflammation and cumulative oxidative stress, which are significant underlying ocular diseases. Thus, AXT is potentially used in a wide array of therapeutic applications. Finally, AXT has a high safety profile, with no adverse events reported in clinical studies [1, 67].

According to Cort and co-workers’ study, AXT was assessed for its suppressive effects on retinal nerve fiber layer injury due to glaucoma. Using mouse models, the researchers unilaterally cauterized episcleral vessels to elevate Intraoptical pressure (IOP). The sample groups were randomly classified into olive oil or an AXT dose of 5 mg/kg/day for eight weeks. In the end, several parameters were evaluated, such as; evoked potential recordings from stimulating visual pathways ‘visual evoked potentials (VEP),’ retinal apoptosis, and expression of oxidative markers to assess AXT’ neuroprotective effects.

As for the group treated with AXT, retinal protein oxidation returned to normal levels after elevated IOP compared to the control group. In addition, all groups with high IOP exhibited higher apoptosis rates than those treated with AXT. A second benefit was the restoration of altered VEP parameters after AXT treatment. Using this method, one can assess changes in the visual system at the earliest stages of an increase in IOP sensitively and reliably [68].

In regard to Dry Eye Syndrome, AXT, In a double-blinded randomized controlled trial, it was found that oral AXT supplementation might inhibit reactive oxygen species (ROS) and enhance tear production [69].

Hence, Data from various non-clinical and clinical investigations indicate the importance of eating a balanced diet for eye health. Interestingly, there is increasing evidence that AXT is presumably beneficial in the prophylaxis and treatment of several ocular diseases, as reviewed elsewhere. Thus, nutraceuticals such as AXT can be prescribed along with other conventional therapies for treating various pathological diseases due to synergistic effects [66].

### Skin health

AXT is presumed to offer skin protection through several mechanisms. According to research, it blocks the absorption of some ultraviolet (UV) radiation that acts directly on the skin. Also, it neutralizes the free radicals induced by UV radiation. Finally, it presumably blocks the activation of matrix metalloproteinase (MMP) induced by UV light, Which is presumed to be a key factor leading to sun damage and skin aging [49].

As highlighted earlier, AXT, a carotenoid, is recognized for its potent antioxidant and anti-inflammatory properties. Biological aging and UV light exposure (photoaging) are the primary contributors to emerging age-related skin changes. Photoaging destroys extracellular matrix components (collagen and elastin), resulting in wrinkles, pigmentation, and degradation of skin texture [67]. Also, UV light induces the skin to release reactive oxygen species. A supplement containing AXT may preserve skin problems caused by environmental damage and prevent age-related skin degradation [70]. Chalyk et al. [74] observed that the oxidative stress indicator, malondialdehyde (MDA), reduced after a human trial participant received a daily dose of 4 mg of AXT. This experimental study showed a reduction of MDA level by 11.2% on the 15th day and 21.7% on the 29th day [70, 71].

### Cardiovascular health

Several studies reported the benefits of AXT to the human body and cardiovascular systems, as it can inhibit the oxidation of low-density lipoproteins and prevent atherosclerosis. The onset of atherosclerosis-related cardiovascular disease depends on oxidative stress and inflammation [72].

Iwamoto and his co-workers studied the potential dose-dependent effects of AXT extracted from krill on plasma samples taken from healthy subjects for a 14-day study. An oxidant agent like V-70 increased the lag time associated with LDL oxidation dose-dependently. In this study, AXT's ability to lower LDL oxidability was more potent than tocopherols and lutein [73].

As hypertension is considered a significant risk factor for cardiovascular diseases, thus, studying the potential benefits of AXT in controlling blood pressure may provide insights into its effects on the cardiovascular system. AXT, when orally administered to the Wistar Kyoto hypertensive rat model 'spontaneously hypertensive rats (SHR),' significantly reduced blood pressure (BP) after 14 days compared to normotensive Wistar Kyoto rats. Also, five weeks of oral administration of AXT to stroke prone SHR have markedly decreased BP, as demonstrated in SHR.' In addition to these results, improvement in nitric oxide-induced vascular relaxation in the aortas of the rats was detected. Orally administered AXT was demonstrated to decrease BP in SHR rats by reducing nitric oxide end products. Also, this is because of its potential effects in decreasing the accumulation of elastin in the aorta and attenuating atherosclerosis by reducing the wall-to-lumen ratio of coronary arteries [74]. There is also growing research evidence supporting the benefits of AXT in other cardiovascular aspects. Inhibiting endothelial cell apoptosis and aging can inhibit cardiovascular disease to a certain extent; vascular endothelial dysfunction and smooth muscle pathological migration mainly occur due to oxidative stress [75].

### Immune response

Human clinical studies studying AXT's potential effects on the immune system were first reported in 2010 by Park et al. During an eight-week controlled, double-blind trial, healthy young female subjects received AXT (0, 2, or 8 mg/day). A dose of 8 mg of AXT increased the cytotoxic activity of natural killer cells, increased the number of T and B lymphocyte subpopulations, and stimulated the proliferation of lymphocytes induced by mitogens, enhancing immunity [32, 33].

This study aimed to evaluate the immunomodulatory effects of AXT on mice and its safety profile. During the experiment, mice were given a low dose of 4.2 mg/kg daily, followed by higher doses (8.35 mg/kg and 16.70 mg/kg) for 30 days. Several immunological parameters were assessed during the analysis, such as the transformation activity of spleen lymphocytes, the delay in an allergic reaction, and the index of the thymus and spleen. In addition, the levels of antibody production, the activity of natural killer cells, the half-hemolytic value, carbon particle clearance rate, and macrophage phagocytosis were also measured. AXT's safety profile was evaluated using acute oral toxicity and genotoxicity. Compared with control groups, medium and high doses of AXT significantly increased the proliferation and transformation of spleen lymphocytes, antibody-producing cells, and serum hemolysin levels. The effects of AXT on delayed allergy reactions and natural killer cell cytotoxicity were also significant.

Furthermore, no signs of acute oral toxicity or genotoxicity were reported. Neither gross anatomical nor histopathological examinations revealed abnormal changes after AXT's treatments. As concluded by this study, AXT was not toxic in experimental doses [35].

Lately, many scientific endeavors are required to control the invasion of viral epidemics, especially in the era of the coronavirus disease 2019 (COVID-19) outbreak. AXT has been studied as an effective strategy to prevent inflammatory effects following COVID-19 due to its potent peroxisome proliferator-activated receptor (PPAR) activity. Jayanta Talukdar and his co-workers found that natural antioxidants, such as AXT, can be therapeutic agents against inflammatory cytokine storms.

## Green nanotechnology

The green synthesis of nanoparticles using biological systems, particularly plant extracts, has become increasingly popular in nanotechnology [76]. Natural plant extracts contain various secondary metabolites and biomolecules, such as flavonoids, alkaloids, terpenoids, phenolic compounds, and enzymes, which enable the reduction of metal ions to nanoparticles in eco-friendly one-step processes. This approach often eliminates the need for stabilizing and capping agents and yields biologically active shape- and size-dependent products [76, 77]. For example, zinc oxide nanoparticles have been synthesized from naturally available eggplant leaf extract or Solanum melongena leaf extract [78]. Previous studies have indicated that an extracellular mechanism for synthesizing silver nanoparticles using bacteria is widely employed. This mechanism involves the reduction of silver ions (Ag^+^) by several bacterial species, such as *Streptomyces sp. LK3* and *Bacillus licheniformis* via nicotinamide adenine dinucleotide hydrogen (NADH)-dependent nitrate reductase-mediated reduction [79]. One study synthesized silver nanoparticles using aqueous extracts of fresh leaves from Impatiens *balsamina* and *Lantana camara* plants. These nanoparticles were comparable to *ciprofloxacin* in inhibiting bacterial growth [80].

Additionally, research has shown that the aqueous extract of *Gundlia tournefortii* leaves is an effective bioreductant in synthesizing gold nanoparticles to treat bacterial, fungal, and skin diseases. This further demonstrates the potential of using biological systems for the green synthesis of nanoparticles and their applications in medicine [81].

The nanoparticles produced are synthesized using various qualitative and quantitative techniques. Qualitative techniques comprise Fourier Transform Infrared Spectroscopy (FT-IR), UV–Vis spectrophotometry, Scanning Electron Microscopy (SEM), X-ray Diffraction (XRD), and Atomic Force Microscopy (AFM). On the other hand, the quantitative techniques consist of Transmission Electron Microscopy (TEM), Annular Dark-Field Imaging (ADF), and Intracranial Pressure (ICP) [82].

## AXT in the nanotechnology era

Innovative AXT-loaded stealth lipid nanoparticles (AXT-SSLNs) were developed to overcome AXT's high stability in clinical practice and to improve AXT bioavailability in the brain. Solvent-diffusion technique was used to prepare AXT-SSLNs suitable for parenteral administration [83].

The stability of AXT was evaluated in a range of carriers and storage settings. It has been studied utilizing microencapsulation with chitosan, polymeric nanospheres, emulsions, and beta-cyclodextrin [5].

### Enhancement of stability and solubility of AXT

To increase AXT's bioavailability, Zhu et al. applied polyethene glycol-grafted chitosan (PEG-g-CS) to AXT using the solvent evaporation method. In an in vitro release assay, AXT that was well-encapsulated by PEG-g-CS (Table 2) was released immediately within 15 min. This formulation increases the oral bioavailability of AXT in vivo and in vitro [84].

**Table 2:** Formulations that increase the stability and solubility of AXT

Formulation	Method of Manufacturing	Characteristics	References
AXT-PEG-g-CS	Solvent Evaporation	Spherical, with a particle size below 200 nm and a ζ potential of about − 26 mV	[84]
α-tocopherol/AXT ascorbic acid/AXT	Emulsification solvent-diffusion method	The particle size of produced astaxanthin nanodispersions (98.3 ± 4.27 nm)	[85]
AXT-PLGA-COS	Anti-solvent precipitation	Smooth spheres with mean particle diameters around 150 nm and positive surface potentials (ζ = + 30 mV)	[22]
AXT-WPC	Emulsification-solvent evaporation	The surface charge (zeta-potential) was (− 20 to − 30 mV), and the particle size ranged from 80 to 130 nm	[86]
Oil-in-water nanoemulsions of AXT	High-pressure homogenization	The mean diameter of the dispersed particles containing AXT ranged from 160 to 190 nm. The dispersion had a zeta potential of less than − 41 mV	[88]
AXT-PCPLC	Solvent displacement method	The diameter of nanoparticles is 300–320 nm. the zeta potential values of − 18.2 and − 29.1 mV	[89]
ADC	Method of macromolecular co-assembly combined with solvent evaporation	AXT content up to 65 µg/ml. The average diameter is 211 nm, and the zeta potential is 35.3 mV	[91]

ADC: Astaxanthin-loaded DNA/chitosan; AXT: Astaxanthin; AXT-PEG-g-CS: Astaxanthin-polyethene glycol-grafted chitosan; AXT-PCPLC: Astaxanthin-poly (ethylene oxide)-4-methoxycinnamoylphthaloyl-chitosan; AXT-PLGA-COS: Astaxanthin-polylactic-co-glycolic acid-chitosan oligosaccharides; AXT-WPC: Astaxanthin-whey protein concentrate

AXT nanodispersions were created using the solvent-diffusion method with the addition of ascorbic acid and α-tocopherol, which can both retard the degradation of AXT. The addition of ascorbic acid (ascorbic acid/AXT w/w) and α-tocopherol (α-tocopherol/AXT w/w) at proportions of 0.4 and 0.6, respectively, would offer AXT the greatest chemical stability, which was measured by using a response surface methodology (RSM) response optimizer. The results showed the superiority of using α-tocopherol to stabilize AXT over ascorbic acid [85].

Poly-lactic-co-glycolic acid (PLGA) nanoparticles coated with chitosan oligosaccharides (COS) (Table 2) were used to encapsulate AXT by the anti-solvent precipitation method. Dynamic light scattering, transmission electron microscopy, and scanning electron microscopy were used to analyze the loaded nanoparticles' characteristics. The results showed the loading capacity (> 15%) and encapsulation efficiency (> 85%) of the comparatively high nanoparticles. The nanoparticles displayed high cytocompatibility, good aqueous solution dispersibility, and stability [22].

Zanoni et al. create a technique to stabilize AXT and enhance its nutritive and functional qualities. They used the emulsification-solvent evaporation approach, where whey protein concentrate (WPC) was a stabilizer, and the encapsulation efficiency was 96%. Whey protein-based nanoparticles that contain AXT had a maximum bio-accessibility of 76%, with 75% of the AXT being converted to the more bioavailable free form [86].

By Whitaker et al., oil-in-water nanoemulsions of AXT were prepared by specific energy methods. The effect of nano-emulsification of AXT on its bioabsorption, degradation, and antioxidant activity was evaluated in different models. It was found that nano-emulsified AXT increased carotenoid plasma concentration up to 7.5-fold compared with the control solution [87].

High-pressure homogenization was used to create oil-in-water nanoemulsions of AXT. The AXT-containing dispersed particles had an average diameter of 160 and 190 nm. The nanoemulsion did not significantly change following one month of storage under light and temperature conditions, showing that the nanoemulsion had a stable colloid with a zeta potential of less than − 41 mV [88].

The freeze-dried AXT-encapsulated with poly (ethylene oxide)-4-methoxycinnamoylphthaloyl-chitosan (PCPLC) nanospheres (Table 2) by using the solvent displacement method showed better dispersibility in water, yielding stable aqueous suspensions of 300–320 nm nanoparticles. Nuclear magnetic resonance (NMR) analysis indicated that after a two-hour heating at 70 ^◦^C in an aqueous environment, PCPLC nano encapsulated AXT showed minimal heat degradation of AXT [89].

Recently, a study evaluated the physicochemical stability and in vitro digestibility of AXT-loaded Pickering emulsions stabilized by two different nanoparticles, zein and Adzuki bean seed coat polyphenol (ABSCP). Using these nanoparticles was an effective delivery system for AXT [90].

### Enhancement of antioxidant activity of AXT

Formulation of AXT-loaded nanostructure lipid carriers using green chemistry and sunflower oil can stabilize and preserve the AXT antioxidant capacity [91]. The AXT-loaded DNA/chitosan (ADC) nanoparticles showed a more potent cytoprotective impact against H2O2-induced oxidative cell damage and increased cell viability. ADC nanoparticles had a ROS scavenging effectiveness of up to 54.3%, two times greater than free AXT. The intestinal epithelial cells could endocytose the encapsulated AXT and absorb it rapidly [92]. The Solid lipid-polymer hybrid nanoparticles (SLPN) were used to encapsulate AXT. After encapsulation in SLPN, the antioxidant activity of AXT was significantly increased in aqueous conditions, and a sustained release was achieved in simulated gastrointestinal (GI) fluids [93].

### AXT-nanoparticle enhancing anticancer activity

#### Blocking inflammatory feed-forward loops

Breast cancer development and metastasis depend on inflammatory feed-forward loops, including the “inflammatory cell recruitment processes.” A doxorubicin micellar low-molecular-weight heparin-AXT nanoparticle (LMWH-AXT/DOX, LA/DOX NP) was created. The nuclear factor-κB (NF-κB) and signal transducer and activator of transcription 3 (STAT3) signalling pathways may be inhibited by the hydrophobic AXT. Therefore LA/DOX NPs can stop these loops by preventing the development of neutrophil extracellular traps (NETs), which prevent breast cancer and limit liver and lung metastasis and reduce the inflammatory and immunosuppressive milieu in tumors (Table 3) [94].

**Table 3:** Formulations of AXT that manage cancer and different other diseases

Formulation	Efficacy	References
LMWH-AXT/DOX	Prevent breast cancer and limit liver and lung metastasis by preventing the development of inflammatory feed-forward loops	[94]
TAP	Prevent Metastatic Lung Melanoma through the TAP antioxidant effect	[95]
PLGA-AXT	Manage skin cancer by increasing cell viability and cellular uptake of AXT	[96]
AXT-lipo	Prevent skin damage due to the antioxidant activity of AXT	[101]
AXT-NPs	Neuroprotective effects on OxyHb-induced neuronal injury	[102]
Chitosan-caseinate-dextran ternary-AXT	Prevent liver fibrosis by lower TGFβ1-induced fibrogenic gene expression level	[103]
PER-AXT-MOMC	AXT and trametinib block myofibroblast activation, and MOMC can repair damaged AEC II to encourage the regeneration of damaged lung tissue	[105]
AXT-GLU-LIP	Treatment of diabetic nephropathy in rats by enhancing renal absorption and bioavailability of AXT	[106]
AXT-TP@KCNE	Management of the complications of diabetes mellitus	[108]

AXT: Astaxanthin; AXT-GLU-LIP: Ligand-modified astaxanthin liposomes; AXT-lipo: Astaxanthin-liposomes; AXT-NPs: Astaxanthin nanoparticles with transferrin; AXT-TP@KCNE: Astaxanthin-alpha-tocopherol with κ-carrageenan nanoemulsion; LMWH-AXT/DOX: doxorubicin micellar low-molecular-weight heparin-astaxanthin nanoparticle; PER-AXT-MOMC: Astaxanthin and trametinib-loaded surface-engineered nanoparticles-monocyte-derived multipotent cells; PLGA-AXT: Poly-lactic-co-glycolic acid-astaxanthin; TAP: D-α-tocopheryl-astaxanthin

#### Apoptosis induction in metastatic lung melanoma

Natural edible peanut oil and D-α-tocopheryl polyethene glycol succinate (TPGS) were combined by Haung et al. to create a stable oil-in-water (O/W) nanoemulsion D-α-tocopheryl that was loaded with AXT (denoted as TAP-nanoemulsion). Otherwise, TAP-nanoemulsion effectively activated the apoptosis pathway in vivo, as the study demonstrated that oral treatment significantly affectselanoma with lung metastases due to the formulation's antioxidant efficacy [95].

#### Treatment of skin cancer

The anti-photodamage effect in immortalized human keratinocytes (HaCaT cells) was examined by Hu et al., who synthesized and improved PLGA nanoparticles loaded with AXT utilizing the emulsion solvent evaporation approach. In vitro research on HaCaT cells showed that the optimized nanoparticles had high cell viability and cellular uptake. The findings indicate that creating PLGA-coated AXT nanoparticles was highly practical and might be used to treat skin conditions or create cosmetic products by reducing ROS levels and restoring mitochondrial membrane potential (Table 3) [96].

### Subarachnoid hemorrhage therapy treatment

A promising way of transferrin conjugated to poly (ethylene glycol) (PEG)-encapsulated AXT nanoparticles (AXT-NPs) on targeted delivery was investigated. The transferrin-containing AXT-NPs exhibited enhanced cellular uptake efficiency than AXT-NPs without transferrin conjugated in primary cortical neurons. Transferrin-containing AXT-NPs with lower AXT concentration showed powerful neuroprotective effects on OxyHb-induced neuronal damage compared to free AXT. These in vitro findings provide insights into the advancement of subarachnoid hemorrhage therapy [97].

### A design of a mitochondria-targeting nanocarrier for enhancing AXT delivery

A novel triphenylphosphonium bromide (TPP)—modified whey protein isolates (WPI)—dextran (DX) conjugates were prepared and characterized for AXT delivery with mitochondria-targeting ability. The TPP–WPI–DX–ASX nanoparticles with spherical shape and nano-size had good stability in stimulated blood. In vitro experiments indicated that the TPP–WPI–DX nanocarriers could effectively realize lysosomal escape and accumulate in the cellular mitochondria. The TPP–WPI–DX–ASX could significantly protect cells against oxidative damage, maintain normal levels of the MMP, and dramatically promote the viability of RAW 264.7 cells [98].

### AXT conjugated polypyrrole nanoparticles for photothermal therapy

Stabilization of AXT by its conjugation with a bovine serum albumin polymer (PPy@BSA-AXT). This novel structure produces many reactive radicals to cause cell death. An experiment using fluorescent microscopic technology showed how photodynamic therapy causes cell death and intracellular organ degradation. As a result, the manufactured PPy@BSA-AXT nanoparticles can be employed as photoacoustic imaging-based prognostic agents for photothermal or photodynamic therapy [99].

### AXT-loaded polymer-lipid hybrid nanoparticles (AXT-LPN) against ototoxicity

One of platinum-based chemotherapy's most common adverse effects, particularly cisplatin therapy, is ototoxicity. There are currently no FDA-approved treatments or preventative measures for this ototoxicity. However, it is commonly accepted that excessive production of ROS in the inner ear causes ototoxicity. AXT is a promising agent for preventing and treating oxidative stress-related diseases [100].

By conjugating AXT and lipid-polymer hybrid nanoparticles (LPN), the solubility of AXT increased; therefore, AXT can pretend round window membranes (RWM) and strongly affect the cisplatin-induced formation of ROS. The results showed that AXT-LPN successfully prevented cells from entering the early stages of apoptosis and restored the decrease in mitochondrial membrane potential caused by cisplatin in vitro [100].

### Protective effects of AXT liposomal on UV-induced skin damage

A practical scavenging ability against singlet oxygen generation in the water phase was demonstrated using chemiluminescence-dependent liposomes carrying AXT. UV-induced skin thickening could be avoided by applying AXT-liposome to the skin before exposure to the sun. Interestingly, preadministration of Asx-lipo also reduced collagen loss brought on by UV exposure. Additionally, topical application of AXT-liposome cationic lipid suppressed melanin synthesis in UV-exposed skin (Table 3) [101].

### AXT-loaded nanoparticles to enhance the neuroprotective effect

In primary cortical neurons, AXT nanoparticles with transferrin conjugated on them (AXT-NPs) demonstrated improved cellular absorption efficiency compared to AXT-NPs without transferrin conjugated. Furthermore, transferrin-containing AXT-NPs with lower AXT concentrations exhibited potent neuroprotective effects on OxyHb-induced neuronal injury compared to free AXT. The increased neuroprotective effects and improved bioavailability made AXT-NPs attractive candidates for AXT's targeted delivery and absorption (Table 3) [102].

### Chitosan-caseinate-dextran ternary complex nanoparticles for potential oral delivery of AXT within liver fibrosis prevention

Stearic acid-chitosan conjugate (SA-CS) and sodium caseinate (NaCas) created biopolymer-based nanoparticles through ionic gelation. Its nanostructure was cross-linked using oxidized dextran (Odex) via Schiff base reaction. The optimized nanoparticles had uniform size distribution and a diameter of 120 nm. High encapsulation efficiency and a good AXT encapsulation capacity with up to a 6% loading ratio were achieved. The radical scavenging ability of the encapsulated AXT was dramatically boosted, which supported the considerable improvement in aqueous dispersibility. The encapsulated AXT displayed drastically increased cellular bioactivity in comparison to the anti-fibrogenic action of free AXT in the human hepatic stellate cell line (LX-2 cells) (Table 3) [103].

### Mesenchymal stem cells vs AXT encapsulated polymerically

Polymeric micelles were created using the synthetic methoxy polyethene glycol-polycaprolactone (mPEG-PCL) copolymer to encapsulate AXT. After that, human mesenchymal stem cells (MSCs) were used to study the impacts on proliferation and differentiation using AXT-loaded polymeric micelles. Results of MSC differentiation revealed that 20 ng/mL polymeric micelles encapsulating AXT increased MSCs' adipogenesis, chondrogenesis, and osteogenesis by 52%, 106%, and 182%, respectively [104].

### Role of AXT to reverse idiopathic pulmonary fibrosis

AXT and trametinib-loaded surface-engineered nanoparticles (PER NPs) are attached to monocyte-derived multipotent cells (MOMC) to create programmed therapies. PER NPs retarget wounded alveolar epithelial cells type II (AEC II) after reacting to matrix metalloproteinase-2 (MMP-2) in Idiopathic pulmonary fibrosis (IPF) tissues. Then, released AXT can boost trametinib's synergistic impact to block myofibroblast activation, and MOMC can also repair damaged AEC II to encourage the regeneration of damaged lung tissue (Table 3) [105].

### AXT natural antioxidant nanosystem for diabetic nephropathy therapy

A novel AXT-based natural antioxidant nanosystem called ligand-modified AXT liposomes (AXT-GLU-LIP) was created and used to treat diabetic nephropathy in rats. It enhanced renal absorption and bioavailability of AXT. Thus AXT showed better performance as an antioxidant agent. As a treatment for diabetic nephropathy, AXT-GLU-LIP may significantly enhance the renal pathological morphology to save the kidney (Table 3) [106].

### AXT-alpha tocopherol nanoemulsion as an anticancer, wound healing, and antibacterial agent

The AXT-alpha tocopherol nanoemulsion (ATNE) was found to be more toxic at higher concentrations than at lower concentrations, according to cytotoxicity assays on three different cancer cells exposed to it for 24 and 48 h. A scratch assay demonstrated the nanoemulsion's ability to heal wounds faster. Significant antibacterial ability to compromise the integrity of the bacterial cell membrane was demonstrated by the minimal inhibitory concentration (MIC) and minimum bactericidal concentrations (MBC) techniques [107].

The characteristics of AXT and alpha-tocopherol for the control of wound healing in people with diabetes have garnered much interest. In comparison to STZ-induced diabetic mice, the AXT and alpha-tocopherol with κ-carrageenan nanoemulsion (AXT-TP@KCNE) demonstrated better management of hyperglycemia and more excellently corrected the problems associated with diabetes mellitus (Table 3) [108].

### Pitfalls and caveats in nanoencapsulation of AXT

The encapsulation of AXT within nanoparticles is associated with several challenges that must be addressed to ensure successful delivery. One of these challenges is the low solubility and instability of AXT. This challenge can be overcome by selecting an appropriate and stable nanocarrier that possesses desirable physicochemical properties and high encapsulation efficiency. Additionally, the use of stabilization agents can help to maintain AXT stability. Another challenge is the variability in AXT bioavailability, which can be addressed by optimizing nanocarrier attributes such as size, shape, and surface charge. The consideration of physiological barriers such as the blood–brain barrier is also crucial. The limited understanding of AXT metabolism necessitates the characterization of AXT metabolites and their effects on bioavailability. Furthermore, interindividual differences in metabolism must be considered. The lack of standardized methods for evaluating AXT bioavailability is another challenge that must be addressed. This requires the development of validated methods for measuring AXT absorption, distribution, metabolism, and excretion. Standardized protocols for preclinical and clinical studies must also be established. The potential toxicity of nanocarriers is another concern that must be addressed. Biocompatible and biodegradable nanocarriers must be selected, and preclinical studies must be conducted to evaluate potential toxicity. Finally, regulatory compliance with nanomaterial guidelines and consideration of patent protection for novel encapsulation methods are essential to address regulatory requirements and intellectual property issues (Table 4) [5, 14, 20, 34–37, 109–113].

**Table 4:** AXT encapsulation challenges and potential solution

Challenge	Potential solution	References
Low solubility and instability of AXT	Selection of stable and appropriate nanocarrier with suitable physicochemical properties and high encapsulation efficiency; use of stabilization agents	[34, 35]
Variability in AXT bioavailability	Optimization of nanocarrier size, shape, and surface charge; consideration of physiological barriers such as the blood–brain barrier	[36, 37]
Limited understanding of AXT metabolism	Characterization of AXT metabolites and their effects on bioavailability; consideration of differences in metabolism between individuals	[5, 20]
Lack of standardized methods for evaluating AXT bioavailability	Development of validated methods for measuring AXT absorption, distribution, metabolism, and excretion; establishment of standardized protocols for preclinical and clinical studies	[109]
Potential toxicity of nanocarriers	Selection of biocompatible and biodegradable nanocarriers; evaluation of potential toxicity in preclinical studies	[110, 111]
Regulatory requirements and intellectual property issues	Compliance with regulatory guidelines for nanomaterials; consideration of patent protection for novel encapsulation methods	[112, 113]

AXT: Astaxanthin

## Conclusions

Due to poor solubility and stability, AXT has limited applications in clinical settings. The double bonds make the molecule liable to oxidation and can transform into different stereoisomers (with trans–cis isomerization). In addition, AXT can be easily degraded by heat and it doesn't dissolve well in water. Therefore, innovative strategies are required to overcome this intriguing natural active compound's limitations and enable its application in the therapeutic sector. It has been found that AXT solubility and stability improved in combination with nanoparticles. Accordingly, several studies have directed their immediate attention to the therapeutic impact of the AXT nanoparticles combination. This article attempts to understand what is new about AXT sources, structures, therapeutic effects, and their applications in the nanotechnology era.

## Data Availability

Data sharing is not applicable to this article as no datasets were generated or analyzed during the current study.

## Abbreviations

ABSCP: Adzuki bean seed coat polyphenol
AD: Alzheimer’s disease
ADC: AXT-loaded DNA/chitosan
ADF: Annular dark-field imaging
AEC II: Alveolar epithelial cells type II
AFM: Atomic force microscopy
Ag^+^: Silver ions
ATNE: AXT-alpha tocopherol nanoemulsion
APP/PSEN1: Amyloid protein precursor/presenilin-1
AXT: Astaxanthin
AXT-DHA: Docosahexaenoic-acid-acylated AXT diesters
AXT-GLU-LIP: Ligand-modified AXT liposomes
AXT-SSLNs: AXT-loaded stealth lipid nanoparticles
BAX: Bcl-2 associated X-protein
Bcl-2: B-cell lymphoma 2
BDN: Brain-derived neurotrophic factor
BP: Blood pressure
BSA: Bovine serum albumin
Cdc2: Cell division cycle 2
COS: Chitosan oligosaccharides
COVID-19: Coronavirus disease 2019
CNS: Central nervous system
DCW: Dry cell weight
DED: Dry eye disease
DNA: Deoxyribonucleic acid
DOX: Doxorubicin
DX: Dextran
EIU: Endotoxin-induced uveitis
FDA: Food and Drug Administration
FT-IR: Fourier transform infrared spectroscopy
GI: Gastrointestinal
GRAS: Generally recognized as safe
HDL: High-density lipoproteins
HMGB1: High-mobility group box 1
ICP: Intracranial Pressure
IL-1: Interleukin-1
IPF: Idiopathic pulmonary fibrosis
IOP: Intraoptical pressure
LDL: Low-density lipoproteins
LMWH: Low-molecular-weight heparin
LPS: Lipopolysaccharide
LPN: Lipid hybrid nanoparticles
MBC: Minimum bactericidal concentrations
MDA: Malondialdehyde
MIC: Minimal inhibitory concentration
MMP: Matrix metalloproteinase
MOMC: Monocyte-derived multipotent cells
mPEG-PCL: Methoxy polyethene glycol-polycaprolactone
MSC: Mesenchymal stem cells
NaCas: Sodium caseinate
NADH: Nicotinamide adenine dinucleotide hydrogen
NETs: Neutrophil extracellular traps
NK: Natural kill
NLC: Nanostructured lipid carrier
NMR: Nuclear magnetic resonance
NO: Nitric oxide
NOS: Nitric oxide synthase
NP: Neuropathic pain
Nrf2: Nuclear factor erythroid 2–related factor 2
Odex: Oxidized dextran
P80: Polysorbate 80
pAKT: Phosphorylated protein kinase B
PCPLC: Poly (ethylene oxide)-4-methoxycinnamoylphthaloyl-chitosan
pCREB: Phosphorylated adenosine 3′5’ cyclic monophosphate-response element binding protein
PD: Parkinson’s disease
PEG-g-CS: Polyethylene glycol-grafted chitosan
pERK: Phosphorylated Protein kinase-like endoplasmic reticulum kinase
PFC: Prefrontal cortex
PGE2: Prostaglandin E2
PLGA: Poly-lactic-co-glycolic acid
PPAR: Peroxisome proliferator-activated receptor
RES: Reticuloendothelial system
ROS: Reactive oxygen species
RSM: Response surface methodology
RWM: Round window membranes
SA-CS: Stearic acid-chitosan
SEM: Scanning electron microscopy
SHR: Spontaneously hypertensive rats
SLNs: Solid lipid nanoparticles
SLPN: Solid lipid-polymer hybrid nanoparticles
STAT3: Signal transducer and activator of transcription 3
STZ: Streptozotocin
TAP: D-α-tocopheryl-astaxanthin
TEM: Transmission electron microscopy
TNF-α: Tumor necrosis factor-alpha
TP@KCNE: Alpha-tocopherol with κ-carrageenan nanoemulsion
TPGS: D-α-tocopheryl polyethylene glycol succinate
TPP: Triphenylphosphonium bromide
UV: Ultraviolet
VEP: Visual evoked potentials
VLDL: Very low-density lipoproteins
WPC: Whey protein concentrate
XRD: X-ray Diffraction

## References

[1] Higuera-Ciapara I, Félix-Valenzuela L, Goycoolea FM (2006). Astaxanthin: a review of its chemistry and applications. Crit Rev Food Sci Nutr.

[2] Pashkow FJ, Watumull DG, Campbell CL (2008). Astaxanthin: a novel potential treatment for oxidative stress and inflammation in cardiovascular disease. Am J Cardiol.

[3] Kidd P (2011). Astaxanthin, cell membrane nutrient with diverse clinical benefits and anti-aging potential. Altern Med Rev.

[4] Guerin M, Huntley ME, Olaizola M (2003). Haematococcus astaxanthin: applications for human health and nutrition. Trends Biotechnol.

[5] Ambati RR, Moi PS, Ravi S, Aswathanarayana RG (2014). Astaxanthin: sources, extraction, stability, biological activities and its commercial applications—a review. Mar Drugs.

[6] Jafari Z, Bigham A, Sadeghi S, Dehdashti SM, Rabiee N, Abedivash A (2022). Nanotechnology-abetted astaxanthin formulations in multimodel therapeutic and biomedical applications. J Med Chem.

[7] Martínez-Álvarez Ó, Calvo MM, Gómez-Estaca J (2020). Recent advances in astaxanthin micro/nanoencapsulation to improve its stability and functionality as a food ingredient. Mar Drugs.

[8] Rao AR, Sindhuja HN, Dharmesh SM, Sankar KU, Sarada R, Ravishankar GA (2013). Effective inhibition of skin cancer, tyrosinase, and antioxidative properties by astaxanthin and astaxanthin esters from the green alga *Haematococcus pluvialis*. J Agric Food Chem.

[9] Basiony M, Ouyang L, Wang D, Yu J, Zhou L, Zhu M (2022). Optimization of microbial cell factories for astaxanthin production: Biosynthesis and regulations, engineering strategies and fermentation optimization strategies. Synth Syst Biotechnol.

[10] Barros MP, Marin DP, Bolin AP, de Cássia Santos Macedo R, Campoio TR, Fineto CJ (2012). Combined astaxanthin and fish oil supplementation improves glutathione-based redox balance in rat plasma and neutrophils. Chem Biol Interact.

[11] Yaqoob Z, Arshad MS, Imran M, Munir H, Qaisrani TB, Khalid W (2022). Mechanistic role of astaxanthin derived from shrimp against certain metabolic disorders. Food Sci Nutr.

[12] Zhang C, Chen X, Too H-P (2020). Microbial astaxanthin biosynthesis: recent achievements, challenges, and commercialization outlook. Appl Microbiol Biotechnol.

[13] Stachowiak B, Szulc P (2021). Astaxanthin for the food industry. Molecules.

[14] Cunningham FX, Gantt E (2007). A portfolio of plasmids for identification and analysis of carotenoid pathway enzymes: *Adonis aestivalis* as a case study. Photosynth Res.

[15] Ide T, Hoya M, Tanaka T, Harayama S (2012). Enhanced production of astaxanthin in Paracoccus sp. strain N-81106 by using random mutagenesis and genetic engineering. Biochem Eng J.

[16] Tramontin LRR, Kildegaard KR, Sudarsan S, Borodina I (2019). Enhancement of astaxanthin biosynthesis in oleaginous yeast yarrowia lipolytica via microalgal pathway. Microorganisms.

[17] Colusse GA, Duarte MER, De-Carvalho JC, Noseda MD, Ravishankar GA, Ranga Rao A (2021). Chapter 7—Production of astaxanthin by *Haematococcus pluvialis*: lab processes to scale up including the cost considerations. Global perspectives on astaxanthin.

[18] Le-Feuvre R, Moraga-Suazo P, Gonzalez J, Martin SS, Henríquez V, Donoso A (2020). Biotechnology applied to *Haematococcus pluvialis* Fotow: challenges and prospects for the enhancement of astaxanthin accumulation. J Appl Phycol.

[19] Villaró S, Ciardi M, Morillas-España A, Sánchez-Zurano A, Acién-Fernández G, Lafarga T (2021). Foods.

[20] Guo J, Jones MJ, Ulrich J (2010). Polymorphism of 3,3’-dihydroxy-β, β-carotene-4,4’-dione (astaxanthin). Chem Eng Res Des.

[21] Osterlie M, Bjerkeng B, Liaaen-Jensen S (1999). Accumulation of astaxanthin all-E, 9Z and 13Z geometrical isomers and 3 and 3’ RS optical isomers in rainbow trout (*Oncorhynchus mykiss*) is selective. J Nutr.

[22] Liu C, Zhang S, McClements DJ, Wang D, Xu Y (2019). Design of astaxanthin-loaded core-shell nanoparticles consisting of chitosan oligosaccharides and poly(lactic-co-glycolic acid): enhancement of water solubility, stability, and bioavailability. J Agric Food Chem.

[23] Jannel S, Caro Y, Bermudes M, Petit T (2020). Novel Insights into the biotechnological production of *Haematococcus pluvialis*-derived astaxanthin: advances and key challenges to allow its industrial use as novel food ingredient. J Mar Sci Eng.

[24] Landon R, Gueguen V, Petite H, Letourneur D, Pavon-Djavid G, Anagnostou F (2020). Impact of astaxanthin on diabetes pathogenesis and chronic complications. Mar Drugs.

[25] Ahmadi A-R, Ayazi-Nasrabadi R (2021). Astaxanthin protective barrier and its ability to improve the health in patients with COVID-19. Iran J Microbiol.

[26] Leung LY-L, Chan SM-N, Tam H-L, Wong ES-W (2022). Astaxanthin influence on health outcomes of adults at risk of metabolic syndrome: a systematic review and meta-analysis. Nutrients.

[27] Visioli F, Artaria C (2017). Astaxanthin in cardiovascular health and disease: mechanisms of action, therapeutic merits, and knowledge gaps. Food Funct.

[28] Okada Y, Ishikura M, Maoka T (2009). Bioavailability of astaxanthin in haematococcus algal extract: the effects of timing of diet and smoking habits. Biosci Biotechnol Biochem.

[29] Zuluaga M, Gueguen V, Letourneur D, Pavon-Djavid G (2018). Astaxanthin-antioxidant impact on excessive reactive oxygen species generation induced by ischemia and reperfusion injury. Chem Biol Interact.

[30] Odeberg JM, Lignell Å, Pettersson A, Höglund P (2003). Oral bioavailability of the antioxidant astaxanthin in humans is enhanced by incorporation of lipid-based formulations. Eur J Pharm Sci.

[31] Singh GKS, Ismail MA, Zulkefli NAA, Mohd Affandi MMRM (2018). Tissue distribution of astaxanthin formulation in rats. Curr Nutr Food Sci.

[32] Park JS, Chyun JH, Kim YK, Line LL, Chew BP (2010). Astaxanthin decreased oxidative stress and inflammation and enhanced immune response in humans. Nutr Metab (Lond).

[33] Donoso A, González-Durán J, Muñoz AA, González PA, Agurto-Muñoz C (2021). Therapeutic uses of natural astaxanthin: an evidence-based review focused on human clinical trials. Pharmacol Res.

[34] McClements DJ (2015). Enhancing nutraceutical bioavailability through food matrix design. Curr Opin Food Sci.

[35] Fan Q, Chen Z, Wu Y, Zhu J, Yu Z (2021). Foods.

[36] Fakhri S, Yosifova Aneva I, Farzaei MH, Sobarzo-Sánchez E (2019). The neuroprotective effects of astaxanthin: therapeutic targets and clinical perspective. Molecules.

[37] Lv C, Dai S, Zang J (2022). Distribution, purification, and delivery of astaxanthin in food system-siftdesk. SDRP J Food Sci Technol.

[38] Lucks S, Muller R. Medication vehicles made of solid lipid particles (solid lipid nanospheres-SLN) 1996.

[39] Mehnert W, Mäder K (2001). Solid lipid nanoparticles: production, characterization and applications. Adv Drug Deliv Rev.

[40] Müller RH, Mäder K, Gohla S (2000). Solid lipid nanoparticles (SLN) for controlled drug delivery—a review of the state of the art. Eur J Pharm Biopharm.

[41] Affandi MMM, Julianto T, Majeed A (2011). Development and stability evaluation of astaxanthin nanoemulsion. Asian J Pharm Clin Res.

[42] Anarjan N, Tan CP (2013). Physico-chemical stability of astaxanthin nanodispersions prepared with polysaccharides as stabilizing agents. Int J Food Sci Nutr.

[43] Hans ML, Lowman AM (2002). Biodegradable nanoparticles for drug delivery and targeting. Curr Opin Solid State Mater Sci.

[44] Gaspar DP, Almeida AJ. Surface-functionalized lipid nanoparticles for site-specific drug delivery. In: Pathak YV, editor. Surface modification of nanoparticles for targeted drug delivery. Springer; 2019. p. 73–98.

[45] Müller RH, Maassen S, Weyhers H, Mehnert W (1996). Phagocytic uptake and cytotoxicity of solid lipid nanoparticles (SLN) sterically stabilized with poloxamine 908 and poloxamer 407. J Drug Target.

[46] Gaspar DP, Faria V, Quintas JP, Almeida AJ (2017). Targeted delivery of lipid nanoparticles by means of surface chemical modification. Curr Org Chem.

[47] Michaelis K, Hoffmann MM, Dreis S, Herbert E, Alyautdin RN, Michaelis M (2006). Covalent linkage of apolipoprotein e to albumin nanoparticles strongly enhances drug transport into the brain. J Pharmacol Exp Ther.

[48] Satoh T, Gupta RC (2016). Chapter 38—Astaxanthin: health benefits and toxicity. Nutraceuticals.

[49] Ekpe L, Inaku K, Ekpe V (2018). Antioxidant effects of astaxanthin in various diseases—a review. J Mol Pathophysiol.

[50] Franceschelli S, Pesce M, Ferrone A, De Lutiis MA, Patruno A, Grilli A (2014). Astaxanthin treatment confers protection against oxidative stress in U937 cells stimulated with lipopolysaccharide reducing O2-production. PLoS ONE.

[51] Li H, Li J, Hou C, Li J, Peng H, Wang Q (2020). The effect of astaxanthin on inflammation in hyperosmolarity of experimental dry eye model in vitro and in vivo. Exp Eye Res.

[52] Ohgami K, Shiratori K, Kotake S, Nishida T, Mizuki N, Yazawa K (2003). Effects of astaxanthin on lipopolysaccharide-induced inflammation in vitro and in vivo. Invest Ophthalmol Vis Sci.

[53] Pereira CPM, Souza ACR, Vasconcelos AR, Prado PS, Name JJ (2021). Antioxidant and anti-inflammatory mechanisms of action of astaxanthin in cardiovascular diseases (review). Int J Mol Med.

[54] Faraone I, Sinisgalli C, Ostuni A, Armentano MF, Carmosino M, Milella L (2020). Astaxanthin anticancer effects are mediated through multiple molecular mechanisms: a systematic review. Pharmacol Res.

[55] Kurihara H, Koda H, Asami S, Kiso Y, Tanaka T (2002). Contribution of the antioxidative property of astaxanthin to its protective effect on the promotion of cancer metastasis in mice treated with restraint stress. Life Sci.

[56] Liu X, Song M, Gao Z, Cai X, Dixon W, Chen X (2016). Stereoisomers of astaxanthin inhibit human colon cancer cell growth by inducing G2/M cell cycle arrest and apoptosis. J Agric Food Chem.

[57] McCall B, McPartland CK, Moore R, Frank-Kamenetskii A, Booth BW (2018). Effects of astaxanthin on the proliferation and migration of breast cancer cells in vitro. Antioxidants (Basel).

[58] Cui L, Li Z, Xu F, Tian Y, Chen T, Li J (2022). Antitumor effects of astaxanthin on esophageal squamous cell carcinoma by up-regulation of PPARγ. Nutr Cancer.

[59] Fakhri S, Abbaszadeh F, Dargahi L, Jorjani M (2018). Astaxanthin: a mechanistic review on its biological activities and health benefits. Pharmacol Res.

[60] Yanai H (2008). Antihypertensive effects of astaxanthin. Integr Blood Press Control.

[61] Chang MX, Xiong F (2020). Astaxanthin and its effects in inflammatory responses and inflammation-associated diseases: recent advances and future directions. Molecules.

[62] Taksima T, Chonpathompikunlert P, Sroyraya M, Hutamekalin P, Limpawattana M, Klaypradit W (2019). Effects of astaxanthin from shrimp shell on oxidative stress and behavior in animal model of Alzheimer’s disease. Mar Drugs.

[63] Bahbah EI, Ghozy S, Attia MS, Negida A, Bin Emran T, Mitra S (2021). Molecular mechanisms of astaxanthin as a potential neurotherapeutic agent. Mar Drugs.

[64] Qiao J, Rong L, Wang Z, Zhang M (2017). Involvement of Akt/GSK3β/CREB signaling pathway on chronic omethoate induced depressive-like behavior and improvement effects of combined lithium chloride and astaxanthin treatment. Neurosci Lett.

[65] Ke Y, Bu S, Ma H, Gao L, Cai Y, Zhang Y (2020). Preventive and therapeutic effects of astaxanthin on depressive-like behaviors in high-fat diet and streptozotocin-treated rats. Front Pharmacol.

[66] Giannaccare G, Pellegrini M, Senni C, Bernabei F, Scorcia V, Francesco A (2020). Clinical applications of astaxanthin in the treatment of ocular diseases: emerging insights. Mar Drugs.

[67] Davinelli S, Nielsen ME, Scapagnini G (2018). Astaxanthin in skin health, repair, and disease: a comprehensive review. Nutrients.

[68] Cort A, Ozturk N, Akpinar D, Unal M, Yucel G, Ciftcioglu A (2010). Suppressive effect of astaxanthin on retinal injury induced by elevated intraocular pressure. Regul Toxicol Pharmacol.

[69] Huang J-Y, Yeh P-T, Hou Y-C (2016). A randomized, double-blind, placebo-controlled study of oral antioxidant supplement therapy in patients with dry eye syndrome. Clin Ophthalmol.

[70] Oslan SNH, Tan JS, Oslan SN, Matanjun P, Mokhtar RAM, Shapawi R (2021). *Haematococcus pluvialis* as a potential source of astaxanthin with diverse applications in industrial sectors: current research and future directions. Molecules.

[71] Chalyk NE, Klochkov VA, Bandaletova TY, Kyle NH, Petyaev IM (2017). Continuous astaxanthin intake reduces oxidative stress and reverses age-related morphological changes of residual skin surface components in middle-aged volunteers. Nutr Res.

[72] Zhang X, Hou Y, Li J, Wang J, Costache V, Morales JG (2021). The role of astaxanthin on chronic diseases. Crystals.

[73] Iwamoto T, Hosoda K, Hirano R, Kurata H, Matsumoto A, Miki W (2000). Inhibition of low-density lipoprotein oxidation by astaxanthin tamami of medicine introduction oxidative modification of low-density lipoprotein (LDL) has been implicated in the pathogenesis of atheroscle-rosis (1, 2). The rapid uptake of oxidative. J Atheroscler Thromb.

[74] Fassett RG, Coombes JS (2012). Astaxanthin in cardiovascular health and disease. Molecules.

[75] Zhang X, Hou Y, Li J, Wang J (2021). The role of astaxanthin on chronic diseases. Crystals (Basel).

[76] Fakhari S, Jamzad M, Kabiri FH (2019). Green synthesis of zinc oxide nanoparticles: a comparison. Green Chem Lett Rev.

[77] El-Seedi HR, El-Shabasy RM, Khalifa SAM, Saeed A, Shah A, Shah R (2019). Metal nanoparticles fabricated by green chemistry using natural extracts: biosynthesis, mechanisms, and applications. RSC Adv.

[78] Nagarajan A, Sethuraman V, Balasubramani V, Sridhar TM, Sasikumar R, Vimala G (2020). Solanum melongena leaf extract-based zinc oxide nanoparticles synthesis using green chemistry concepts. IJCA.

[79] Ratan ZA, Haidere MF, Nurunnabi Md, Shahriar SMd, Ahammad AJS, Shim YY (2020). Green chemistry synthesis of silver nanoparticles and their potential anticancer effects. Cancers (Basel).

[80] Aritonang HF, Koleangan H, Wuntu AD (2019). Synthesis of silver nanoparticles using aqueous extract of medicinal plants’ (*Impatiens balsamina* and *Lantana camara*) fresh leaves and analysis of antimicrobial activity. Int J Microbiol.

[81] Zhaleh M, Zangeneh A, Goorani S, Seydi N, Zangeneh MM, Tahvilian R (2019). In vitro and in vivo evaluation of cytotoxicity, antioxidant, antibacterial, antifungal, and cutaneous wound healing properties of gold nanoparticles produced via a green chemistry synthesis using *Gundelia tournefortii* L. as a capping and reducing agent. Appl Organomet Chem.

[82] Ijaz I, Gilani E, Nazir A, Bukhari A (2020). Detail review on chemical, physical and green synthesis, classification, characterizations and applications of nanoparticles. Green Chem Lett Rev.

[83] Santonocito D, Raciti G, Campisi A, Sposito G, Panico A, Siciliano EA (2021). Astaxanthin-loaded stealth lipid nanoparticles (AST-SSLN) as potential carriers for the treatment of Alzheimer’s disease: formulation development and optimization. Nanomaterials (Basel).

[84] Zhu Y, Gu Z, Liao Y, Li S, Xue Y, Firempong MA (2022). Improved intestinal absorption and oral bioavailability of astaxanthin using poly (ethylene glycol)-graft-chitosan nanoparticles: preparation, in vitro evaluation, and pharmacokinetics in rats. J Sci Food Agric.

[85] Anarjan N, Nehdi IA, Tan CP (2013). Protection of astaxanthin in astaxanthin nanodispersions using additional antioxidants. Molecules.

[86] Zanoni F, Vakarelova M, Zoccatelli G (2019). Development and characterization of astaxanthin-containing whey protein-based nanoparticles. Mar Drugs.

[87] Domínguez-Hernández CR, García-Alvarado MA, García-Galindo HS, Salgado-Cervantes MA, Beristáin CI (2016). Stability, antioxidant activity and bioavailability of nano-emulsified astaxanthin. Rev Mex Ing Quim.

[88] Kim D-M, Hyun S-S, Yun P, Lee C-H, Byun S-Y (2012). Identification of an emulsifier and conditions for preparing stable nanoemulsions containing the antioxidant astaxanthin. Int J Cosmet Sci.

[89] Tachaprutinun A, Udomsup T, Luadthong C, Wanichwecharungruang S (2009). Preventing the thermal degradation of astaxanthin through nanoencapsulation. Int J Pharm.

[90] Ge S, Jia R, Li Q, Liu W, Liu M, Cai D (2022). Pickering emulsion stabilized by zein/Adzuki bean seed coat polyphenol nanoparticles to enhance the stability and bioaccessibility of astaxanthin. J Funct Foods.

[91] Rodriguez-Ruiz V, Salatti-Dorado JÁ, Barzegari A, Nicolas-Boluda A, Houaoui A, Caballo C (2018). Astaxanthin-loaded nanostructured lipid carriers for preservation of antioxidant activity. Molecules.

[92] Wang Q, Zhao Y, Guan L, Zhang Y, Dang Q, Dong P (2017). Preparation of astaxanthin-loaded DNA/chitosan nanoparticles for improved cellular uptake and antioxidation capability. Food Chem.

[93] Wang T, Hu Q, Lee J-Y, Luo Y (2018). Solid lipid-polymer hybrid nanoparticles by in situ conjugation for oral delivery of astaxanthin. J Agric Food Chem.

[94] Lu Z, Long Y, Li J, Li J, Ren K, Zhao W (2021). Simultaneous inhibition of breast cancer and its liver and lung metastasis by blocking inflammatory feed-forward loops. J Control Rel.

[95] Haung H-Y, Wang Y-C, Cheng Y-C, Kang W, Hu S-H, Liu D (2020). A novel oral astaxanthin nanoemulsion from *Haematococcus pluvialis* induces apoptosis in lung metastatic melanoma. Oxid Med Cell Longev.

[96] Hu F, Liu W, Yan L, Kong F, Wei K (2019). Optimization and characterization of poly (lactic-co-glycolic acid) nanoparticles loaded with astaxanthin and evaluation of anti-photodamage effect in vitro. R Soc Open Sci.

[97] Zhang X-S, Zhang X, Zhou M-L, Zhou X-M, Li N, Li W (2014). Amelioration of oxidative stress and protection against early brain injury by astaxanthin after experimental subarachnoid hemorrhage. J Neurosurg.

[98] Chen Y, Tie S, Zhang X, Zhang L, Tan M (2021). Preparation and characterization of glycosylated protein nanoparticles for astaxanthin mitochondria targeting delivery. Food Funct.

[99] Bharathiraja S, Manivasagan P, Oh Y-O, Moorthy MS, Seo H, Bui NQ (2017). Astaxanthin conjugated polypyrrole nanoparticles as a multimodal agent for photo-based therapy and imaging. Int J Pharm.

[100] Gu J, Chen Y, Tong L, Wang X, Yu D, Wu H (2020). Astaxanthin-loaded polymer-lipid hybrid nanoparticles (ATX-LPN): assessment of potential otoprotective effects. J Nanobiotechnol.

[101] Hama S, Takahashi K, Inai Y, Shiota K, Sakamoto R, Yamada A (2012). Protective effects of topical application of a poorly soluble antioxidant astaxanthin liposomal formulation on ultraviolet-induced skin damage. J Pharm Sci.

[102] You Z-Q, Wu Q, Zhou X-M, Zhang X-S, Yuan B, Wen L-L (2019). Receptor-mediated delivery of astaxanthin-loaded nanoparticles to neurons: an enhanced potential for subarachnoid hemorrhage treatment. Front Neurosci.

[103] Hu Q, Hu S, Fleming E, Lee J-Y, Luo Y (2020). Chitosan-caseinate-dextran ternary complex nanoparticles for potential oral delivery of astaxanthin with significantly improved bioactivity. Int J Biol Macromol.

[104] Zhang J, Peng C-A (2019). Enhanced proliferation and differentiation of mesenchymal stem cells by astaxanthin-encapsulated polymeric micelles. PLoS ONE.

[105] Chang X, Xing L, Wang Y, Yang C-X, He Y-J, Zhou T-J (2020). Monocyte-derived multipotent cell delivered programmed therapeutics to reverse idiopathic pulmonary fibrosis. Sci Adv.

[106] Chen Z, Li W, Shi L, Jiang L, Li M, Zhang C (2020). Kidney-targeted astaxanthin natural antioxidant nanosystem for diabetic nephropathy therapy. Eur J Pharm Biopharm.

[107] Shanmugapriya K, Kim H, Saravana PS, Chun B-S, Kang HW (2018). Astaxanthin-alpha tocopherol nanoemulsion formulation by emulsification methods: Investigation on anticancer, wound healing, and antibacterial effects. Colloids Surf B Biointerfaces.

[108] Shanmugapriya K, Kim H, Kang HW (2019). A new alternative insight of nanoemulsion conjugated with κ-carrageenan for wound healing study in diabetic mice: In vitro and in vivo evaluation. Eur J Pharm Sci.

[109] Madhavi D, Kagan D, Seshadri S (2018). A study on the bioavailability of a proprietary, sustained-release formulation of astaxanthin. Integr Med A Clin J.

[110] Bahadar H, Maqbool F, Niaz K, Abdollahi M (2016). Toxicity of nanoparticles and an overview of current experimental models. Iran Biomed J.

[111] Zeb A, Rana I, Choi H-I, Lee C-H, Baek S-W, Lim C-W (2020). Potential and applications of nanocarriers for efficient delivery of biopharmaceuticals. Pharmaceutics.

[112] Mohajerani A, Burnett L, Smith JV, Kurmus H, Milas J, Arulrajah A (2019). Nanoparticles in construction materials and other applications, and implications of nanoparticle use. Materials.

[113] Istiqola A, Syafiuddin A (2020). A review of silver nanoparticles in food packaging technologies: regulation, methods, properties, migration, and future challenges. J Chin Chem Soc.

