# Correction: Production and therapeutic use of astaxanthin in the nanotechnology era

**DOI:** 10.1007/s43440-023-00502-3

**Published:** 2023-06-13

**Authors:** Karim Abdelazim, Amr Ghit, Dina Assal, Neamat Dorra, Nehad Noby, Sherine N. Khattab, Shaymaa Essam El Feky, Ahmed Hussein

**Affiliations:** 1 Department of Biotechnology, Institute of Graduate Studies and Research, Alexandria University, Alexandria, Egypt; 2 Department of Medicine and Aging Sciences, “G. d’Annunzio” University of Chieti-Pescara, Chieti, Italy; 3 Center for Advanced Studies and Technology, “G. d’Annunzio” University of Chieti-Pescara, Chieti, Italy; 4 Department of Biology, Biotechnology Program, American University in Cairo, Cairo, Egypt; 5 Department of Microbiology and Immunology, Faculty of Pharmacy, Sinai University-Kantara Branch, Ismailia, Egypt; 6 Chemistry Department, Faculty of Science, Alexandria University, Alexandria, Egypt; 7 Radiation Sciences Department, Medical Research Institute, University of Alexandria, Alexandria, Egypt

**Correction: Pharmacological Reports** 10.1007/s43440-023-00488-y

In this article the affiliation for Neamat Dorra was incorrectly given. It should have been "Department of Microbiology and Immunology, Faculty of Pharmacy, Sinai University-Kantara Branch, Ismailia, Egypt".

The original article has been corrected.

